# Cell Migration and Invasion Assays as Tools for Drug Discovery

**DOI:** 10.3390/pharmaceutics3010107

**Published:** 2011-03-11

**Authors:** Keren I. Hulkower, Renee L. Herber

**Affiliations:** Platypus Technologies, LLC, 5520 Nobel Drive, Suite 100, Madison, WI 53711, USA

**Keywords:** cell migration, cell invasion, high throughput screening, high content analysis, phenotypic assays, drug discovery, 3-dimensional assays, wound healing, cancer metastasis

## Abstract

Cell migration and invasion are processes that offer rich targets for intervention in key physiologic and pathologic phenomena such as wound healing and cancer metastasis. With the advent of high-throughput and high content imaging systems, there has been a movement towards the use of physiologically relevant cell-based assays earlier in the testing paradigm. This allows more effective identification of lead compounds and recognition of undesirable effects sooner in the drug discovery screening process. This article will review the effective use of several principle formats for studying cell motility: scratch assays, transmembrane assays, microfluidic devices and cell exclusion zone assays.

## 1. Introduction

Cell migration is defined as the movement of individual cells, cell sheets and clusters from one location to another [1,2]. It is central to a variety of different pathologic and physiologic processes across many disciplines of biology including wound healing, cancer, inflammation, cell growth and differentiation [3,4]. Cell invasion refers to 3-dimensional migration of cells as they penetrate an extracellular matrix (ECM) and is a process typically associated with cancer cell metastasis [2].

The migration process in wound healing is coordinated among several different cell types including keratinocytes, fibroblasts, endothelial cells and macrophages that provide a variety of growth factors. Platelet derived growth factor (PDGF-BB), basic fibroblast growth factor (bFGF) and granulocyte macrophage colony stimulating factor (GM-CSF) are used as stimulants to facilitate wound healing for patients with diabetic ulcers [5]. These growth factors, however, have had only a moderate clinical effect when used as single agent therapies. Thus, researchers are continuing to probe mechanisms of action in skin progenitor cells to gain a better understanding toward identifying therapies to promote cell migration in wound healing [6].

Similar to the complex mechanisms underlying the process of wound healing, many different factors are also involved in cancer cell metastasis. There are diverse mechanisms that cells can employ to initiate and progress invasion and each offers specific pharmacologic targets for development of anti-metastatic therapies [7]. In order to metastasize through surrounding tissue, tumor cells employ mechanisms that utilize matrix metalloproteases (MMP) and urokinase plasminogen activator (uPA) to digest the ECM. Conversely, cyclooxygenase-2 inhibition has been suggested to block angiogenesis, a mechanism which supports the growth of primary tumors and enables distal metastasis [8]. Monotherapy using synthetic protease inhibitors was not efficacious as cancer therapy in human clinical trials [9]. It was subsequently shown that protease-independent mechanisms exist that tumor cells employ in order to migrate through ECM at undiminished rates [10-13] that could explain lack of efficacy of monotherapy with MMP inhibitors. A combination of inhibitors that address several facets of tumor growth and metastasis continues to be a promising concept for therapy. Indeed, a mixture of the uPA inhibitor WX-UK1, the MMP inhibitor galardin and the cyclooxygenase-2 inhibitor celecoxib was shown to be synergistic in successfully blocking tumor cell migration and invasion *in vitro* [8]. Therefore, high throughput screening (HTS) could be harnessed to more efficiently and effectively test combinations of inhibitors for synergy against phenotypic targets in physiologically relevant cell-based assays for both wound healing and cancer cell metastasis.

The goal of HTS is to accelerate the drug discovery process by rapidly evaluating large compound libraries [14]. To achieve rapid screening and generation of results, HTS uses a combination of robotic processing and high density, low volume assay formats such as found in 384- and 1536-well plates. These approaches minimize testing costs by reducing the amounts of test compound and reagent used. Homogeneous assays requiring only additions and incubations followed by a final read step are the preferred format for HTS because they reduce the robotic complexity requirements for automation [14]. Equally critical for rapid compound analysis is the availability of high throughput detection systems that maximize the utility of these screens. Cell-based assays have recently gained popularity over biochemical assays due to a wider variety of optical detection methods that enable information-rich, multiparameter readouts using live cells in real-time [15]. Zock [16] describes high content screening (HCS) as the application of automated microscopy and image analysis to drug discovery and cell biology in order to permit the accurate capture, measurement and reporting of cellular phenotypes by individually assessing a multitude of attributes. High-content imaging systems provide vast amounts of information with minimal additional cost per assay point where data analysis is often automated by image algorithms that outline cells and define the amount of signal in the identified objects [17]. Advances in instrumentation and computation have firmly placed imaged-based assays in the HTS domain, where modern HCS instruments can image and analyze tens of thousands of assay wells per day.

Justice *et al.* [18] assessed current cell-based assay screening paradigms and stated: “Within five years, decreasing capital investment costs and improved software will make HCS the industry standard for drug screening. Several factors will drive the adoption of high content screens, including the ability to perform multidimensional and multiplexed assays generating *in vivo*-like data for all segments of the drug discovery pipeline, such as target validation, screening and toxicology.” Similarly, Carragher [19] points out that advances in automated cell culture that combine a quick screen for 3-dimensional invasion with the ability to subsequently investigate drug mode of action, including cytotoxicity, for screening small libraries of chemical therapeutics will have the potential to influence decision making early in drug discovery and improve product development time. Drug discovery researchers recognize the need for *in vitro* assays that employ 3-dimensional matrices to provide relevant microenvironments for cellular studies [4,10]. Indeed, the behavior of normal and malignant cells can be distinguished morphologically in 3-dimensional environments [20]. As a consequence, a paradigm shift has begun that calls for development of cell assay models that are more predictive of *in vivo* mechanisms of invasion [21]. Such models that allow elucidation of the mechanisms of migration and invasion are better suited for screening pharmacological compounds than biochemical assays performed in cell lysates [7,19].

In this article, we review the different assay formats employed to study cell motility. For many years, Boyden chamber based transmembrane assays and scratch wound assays were the only widely available formats to study cell migration and invasion. However, new technologies such as microfluidic chambers and exclusion zone assays have recently emerged as alternative phenotypic screening assays that provide additional or complementary information to researchers interested in high content analysis. The advantages and optimal utility of these formats will be covered with specific examples provided for each.

## 2. Scratch Assays

**Figure 1.**
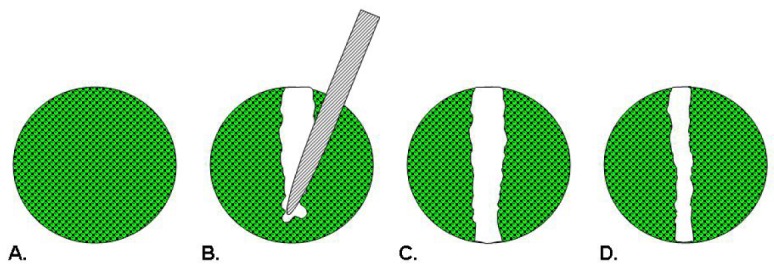
Scratch Assay. A wound is introduced into a confluent monolayer of cells (**A**) by drawing a tip across the cell layer (**B**). The denuded area is imaged to measure the boundary of the wound at pre-migration (**C**) and after cells have migrated inward to fill the void (**D**).

Scratch assays were first used as models of wound healing for epithelial or mesenchymal cells [22]. In this assay depicted in Figure 1, cells are seeded into a multiwell assay plate and allowed to attach, spread, and form a confluent monolayer. A pin tool or needle is used to scratch and remove cells from a discrete area of the confluent monolayer to form a cell-free zone into which cells at the edges of the wound can migrate [22,23]. Molecules of interest as potential therapeutics are added to the wells and images of cell movement are captured at regular intervals within a 24 hour period for data analysis [23]. Essen BioScience has commercialized the CellPlayer™ Migration Assay that offers label-free, kinetic readouts. This assay employs an automated wound maker tool, dedicated 96-well plates and software requiring a capital investment and uses specialized live-cell imaging instrumentation [24].

A majority of researchers employing the scratch assay method utilize multiwell plates that contain 96 or fewer wells. Yarrow *et al.* designed a 384-well format scratch assay using a 96-head pin tool array to scratch cell monolayers [23]. They identified a novel Rho-kinase inhibitor after using this image-based HTS 384-well scratch assay to screen a library of 16,000 small molecules. After seven hours of compound treatment, they were able to observe five predominant phenotypes that addressed mechanisms of action and revealed cytotoxic effects. Based on the results of this HTS primary screen, labor intensive secondary screening was thus limited to a manageable subset of 200 compounds [25].

Simpson *et al.* [26] screened over 1000 siRNA molecules for their ability to regulate MCF-10A breast epithelial cell migration where cells were seeded in 96-well plates, transfected with siRNA molecules and incubated for 58 hours. The monolayer was scratched using a robotically driven pin tool and after 12 hours, migration was analyzed. The effect of siRNA knockdown on cell viability was scored using Alamar blue staining and closure data was obtained via automated fluorescence microscopy of rhodamine conjugated phalloidin stained cells with image analysis of open area using MetaXpress® software. This phenotypic screen enabled the researchers to identify genes that had no impact on cell migration, accelerated cell migration or impaired closure of the scratch. Interestingly, the Z′-factors calculated for this assay indicated that it is more robust when used to identify siRNA molecules that inhibit cell migration (Z′ = 0.41) than those that accelerate migration (Z′ = 0.29) [26,27].

Kam *et al.* describes the inherent limitations of scratch assays as an inability to achieve reproducible and quantitative results [28]. To address this issue, they describe a novel approach to wound healing assays that utilizes a silicone-tipped drill press to create uniformly sized circular wounds in an intact confluent monolayer of cells in 35 mm dishes. They further describe the subsequent addition of a Matrigel™ overlay to the assay wells in order to create an invasion format of the assay. The degree of wound closure or invasion was determined using time-lapse microscopy and ImageJ analysis to measure percent closure of the wounded area within the captured images [28]. The *in vitro* results distinguished different levels of aggressiveness and invasiveness for three different cell lines which corresponded to their behavior *in vivo* [28].

Scratch assays provide several distinct advantages in that: (1) the assay can be performed in any readily available plate configuration; (2) cells move in a defined direction, *i.e.*, to close the wound; (3) the assay surface can be coated with an ECM of choice prior to the experiment; and (4) the movement and morphology of the cells can be visually observed in real time and images captured throughout the experiment thereby permitting velocity measurements [29]. However, there are some drawbacks of this system. As this assay is a “home-brew”, the methods for creating the scratches vary among different labs [29] and the size, shape, and spacing of the scratches can vary from assay well to assay well within a given experiment [28-30]. Moreover, it is difficult to ensure that control and experimental treatment groups are run under equivalent conditions of monolayer confluence and that the area of denudation to form the wound is precise [30]. The process of scratching the monolayer also has been shown to damage the underlying ECM [28,29] and the results may be compromised by the release of factors from the damaged cells [30]. Both laser photoablation [31] and electrical wound assays [32] exist as alternative techniques to remove cells from a confluent monolayer along a specified geometry to overcome the limitations of scratch-wounding. ECIS, commercially available from Applied Biophysics, employs electrical signals to create a wound and monitor cell motility [33]. While ECIS is a fully automated assay, it requires dedicated instrumentation and plates embedded with electrodes to measure transendothelial electrical resistance/impedance of adherent cells. Although these techniques may create well defined wounds, there is still potential for confounding results due to contributions from the destroyed or injured cells near the wound border [34]. Additionally, cell fragments remain on top of the electrodes used to apply the voltage to wound the cells and the damage done to the adjacent cells is unknown [34].

## 3. Transmembrane Assays

**Figure 2.**
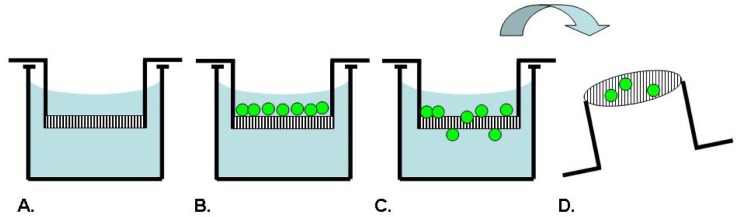
Transmembrane/Boyden Chamber Assay. A membrane insert is used to establish 2 compartments in a well (**A**). Cells are added to the upper compartment (**B**) and migrate through the membrane (**C**). Cell migration is measured by counting the number of cells on the underside of the membrane (**D**). Assay options include coating the membrane with a matrix protein and adding a chemoattractant to the lower compartment.

Boyden originally described an assay technique as depicted in Figure 2, which uses a chamber separated into two compartments by a porous filter membrane to allow the study of leukocyte migration in response to antibody-antigen complexes as a chemotactic agent [35]. In this assay, the cells are seeded on one side of the membrane, while a solution to be tested for chemotactic activity is placed on the opposing side. After an incubation period, the membrane is fixed and stained. The number of cells which have migrated through the pores to the underside of the membrane in response to the chemotactic agent is counted microscopically [35]. Inhibitors of chemotaxis can be tested for activity by including them in one or both of the assay compartments. This assay has been broadly commercialized by several manufacturers such as BD Biosciences, Costar and R&D Systems as product lines of disposable transmembrane inserts for 24- or 96-well tissue culture plates. The membranes are available with different pore sizes to accommodate different cell types and with several choices of ECM coatings for use as a model of invasion [4].

Ogasawra and co-workers [36] utilized Costar Transwell cell culture chambers in a 24-well format to screen 75 types of natural compounds from a variety of chemical classes for their ability to inhibit migration of the murine colon adenocarcinoma cell line 26-L5. The undersides of filters with 8 μm pores were coated with fibronectin and colon 26-L5 cells were pretreated with agents for 30 minutes prior to adding them to the upper compartment. Agents were added to the lower compartment at the same concentrations and after a 3 hour incubation to allow for migration, the cells were fixed and stained with 0.5% crystal violet for 30 minutes. Cells that migrated to the lower surfaces of the filters were acid extracted and absorbance at 590 nm was measured as a means of quantification. They found 23 natural compounds which significantly inhibited tumor cell migration at IC_50_ values 20-fold less than that needed to abolish cell proliferation [36]. However, since the cells were chemically extracted from the membrane, no phenotypic effects of the inhibitors could be observed.

In an effort to improve sensitivity and throughput of transmembrane assays, Mastyugin *et al.* [37] used a modified Boyden assay stained with Hoechst and analyzed on a Cellomics HCS ArrayScan®. The BD BioCoat™ Angiogenesis System that was used is composed of a BD FluoroBlok™ insert plate containing a fibronectin coated fluorescence-blocking microporous membrane in a 96-well format. Human umbilical vein endothelial cells (HUVEC) migrated in response to vascular endothelial growth factor (VEGF) or fetal bovine serum (FBS) added to the lower compartment as the chemoattractant in an 8 hour assay. Migration was quantified by cell enumeration (Hoechst) or fluorescence intensity (Calcein AM), using the ArrayScan®. A 10-fold improvement in the assay signal-to-background was observed by enumerating cells using the Hoechst nuclear stain compared to determining fluorescence intensity using Calcein AM staining. Additionally, the authors were able to discern the chemotactic effect of VEGF versus that of FBS in this assay and demonstrated a dose-dependent effect of the VEGF tyrosine kinase inhibitor AAL993 on endothelial cell migration. The assay readout for a 96-well plate required 65 minutes where nine fields were captured per well with a maximum throughput of 2,000 wells/day [37]. While the assay required less hands-on time, the authors pointed out that this assay is limited as the chemotactic gradient is steep and only transiently established, there is no control over the rate of cell migration, only 15–25% of the cells cross the membrane in the 8 hour assay, and monitoring cell migration in real time is still technically complicated. Their assay yielded nominally acceptable Z′ factors of 0.3–0.4 [37].

An alternative assay that uses a measurement of transepithelial electrical resistance (TEER) to indicate the integrity and permeability of cell monolayers was described by Mandic *et al.* [38]. In this assay, the MDCK-C7 epithelial cell line was grown on porous filter membranes. A defined number of cells from different squamous cell carcinoma lines was applied on top of the MDCK-C7 monolayers and the TEER was measured using a voltmeter. Differences in TEER values over a 3–5 day period, as measured by electrical resistance, were obtained after the addition of tumor and control cells. The TEER assay was capable of demonstrating differences in invasiveness between different head and neck squamous cell carcinoma lines [38].

Transmembrane assays offer the distinct advantages of being able to analyze migration in response to a chemotactic gradient and can be used with adherent as well as non-adherent cells. Filter membranes may be coated with ECM proteins in an effort to better approximate physiological conditions. However, there are several drawbacks of these assays in that they are technically challenging to set up, the gradient is non-linear and equilibrates between both compartments over time [4], it is difficult to visualize the cells during the experiments, and the cells must migrate through a non-physiologic polycarbonate or polypropylene filter. Care must be taken to prepare a single cell suspension of cells for loading in the transmembrane assays. Under-trypsinization can lead to cell clumping while over-trypsinization could strip off adhesion molecules necessary for migration [4]. It is difficult to obtain accurate and statistically significant results when only small numbers of cells cross the filter or when the distribution and/or staining of the cells is uneven [30]. The use of automated imaging and software has been an improvement to counting by eye, however, the software is not always able to discern between the pores of the filter and the cells themselves [30].

## 4. Microfluidic Chamber Assays

**Figure 3.**
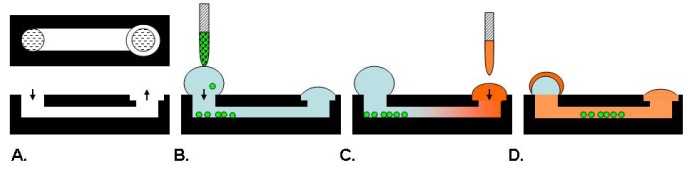
Microfluidic Assay. The device provides 2 ports for reagent delivery (**A**). Cells alone or in a matrix are introduced into the smaller port and adhere to the chamber bottom (**B**). Test agents are added to the larger port and a gradient sets up based on surface tension (**C**). Cells can be imaged to measure migration in response to the test agent (**D**).

Microfluidic systems such as microarrays, gradient devices, valved arrays and individually addressable channel arrays have recently emerged with the potential to be physiologically relevant and improve assay content to provide cell microenvironments for drug discovery [39]. An example schematic is provided in Figure 3. Specifically, a 192 individually addressable microchannel array device was developed at BellBrook Labs which uses droplet-based passive pumping to seed cells, exchange media, and then fix and stain cells [39,40]. This device can be fully automated in conjunction with liquid handling and microscopic imaging instruments [40].

A microfluidic assay was used to demonstrate tumor cell migration through 3-dimensional matrices [40]. In this assay, the microchannels were filled with either a rat tail collagen I or Matrigel™ which was allowed to gel prior to the addition of 2,000 PC-3M cells in a 2.5 μL droplet. After an incubation period to allow the cells to adhere to the ECM, an additional 2.5 μL droplet of media containing test compounds was added and the assay device was incubated in a humidified bioassay dish for up to five days with daily exchanges of media. Cells were fixed and stained with either Alexa 594-conjugated phalloidin or Hoechst 33342 prior to imaging. Images were captured using a microscope with an automated stage and camera in conjunction with Metamorph® software for image analysis and cell counts. A Z′ factor of 0.44 was calculated for this assay as determined by treatment of cells with 5 μM blebbistatin as a pre-invasion control compared with untreated controls permitted to invade in the absence of compounds [40].

Microfluidic chamber based assays can offer distinct advantages in situations where reagent availability is limited. A 800 nL volume of matrix is required to fill channels and only 1,000–2,000 cells are dispensed per channel which represents a 10–100 fold reduction from other methods without sacrificing assay robustness and better enabling the use of rare primary cells [40]. However, fabrication challenges exist to develop interfaces which allow for easy assay automation and handling [39]. Because of the minute volumes, the microfluidics assay requires daily exchanges of growth media leading to increased hands-on time and labor cost involved in the assay [40]. Additionally, care must be taken to maintain a humid local environment surrounding the microchannel plates to avoid evaporation [40]. Materials to fabricate these devices must not have any undesirable effects on absorption of small hydrophobic molecules which are critical in controlling cell microenvironments and must be able to be sterilized [39]. The materials must also be chosen carefully for cost effectiveness in product development and for disposability after single use [39]. Meyvantsson and Beebe [39] presciently acknowledge that microfluidic devices will not be adopted by researchers unless they are facile enough to be used without the aid of engineers.

## 5. Cell Exclusion Zone Assays

Cell exclusion zone assays originated from the need to study cell migration on an uncompromised surface uncoupled from contributions of cell damage and permeabilization that can arise from scratch wounds [34]. Poujade *et al.* explain: “The scratch process destroys the removed cells, which release their intracellular content into the medium; this process is also quite traumatic for the cells on the newly formed border. Indeed these border cells may become partially permeable as a result of the brutal tearing off of the adhesive junctions they maintain with their neighbors” [34]. Thus, they designed a “model wound” assay system by fabricating an elastomeric microstencil mask that is placed in contact with a well bottom and used to pattern adherent cells in parallel lines in the well. Upon reaching confluence, the microstencil mask was removed allowing the cells to collectively migrate on the newly revealed surfaces. They were able to obtain regular, well defined wounds of perfectly controlled width with no cell debris, giving an absolute demarcation point from which to measure migration. In addition, they were able to coat the wells with fibronectin prior to application of the stencil [34].

**Figure 4.**
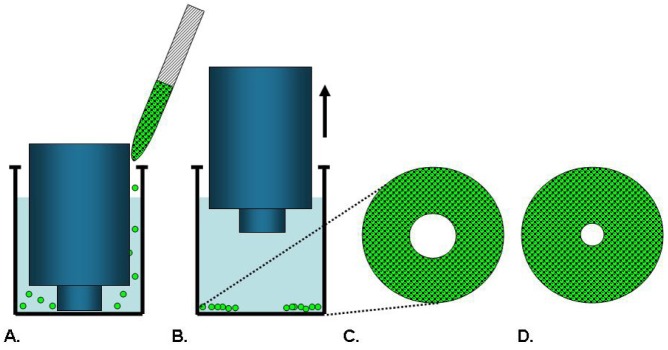
Cell Exclusion Zone Assay. Cells are seeded around a barrier (**A**) and adhere to the well bottom. The barrier is removed (**B**) to reveal a void available for cell movement. The cells are imaged at pre-migration (**C**) and after cells have migrated inward to fill the void (**D**). Assay options include coating the assay well with a matrix and adding an overlay of matrix to create a 3-dimensional assay.

Cell exclusion zone assays, as illustrated in Figure 4, are commercially available from Platypus Technologies and Cell Biolabs. Platypus′ Oris™ Cell Migration Assay uses a 96-well plate populated with silicone-based cell seeding stoppers which exclude cells from attaching to a central zone. After the cells are seeded and allowed to adhere, the stoppers are removed to reveal a 2 mm diameter exclusion zone into which cells may then migrate [29,41]. Stoppers remain in designated wells until assay readout to serve as pre-migration references. Cell migration data may be captured from the Oris™ stopper-based assays using a variety of different methods including microplate readers, microscopes, and high content imaging instruments [29]. This stopper-based assay format allows cells to be transfected with either miRNA or siRNA reagents directly in the wells after cells are seeded. The cells are allowed to recover for 24 hours after transfection before the stoppers are removed to initiate migration of the transfected cells into the detection zone [42]. Modifications of this assay provide plates that are not pre-populated with stoppers and permit the coating of specific ECM proteins or fragments. Once the assay plates are coated, the stoppers are inserted and the cells are added. Using this method, it was determined that, among human fibronectin proteins, the third type III module mediates the migration of human dermal fibroblasts [43].

The Oris™ Cell Migration Assay was validated for HTS using 3-day plate uniformity and replication of potency protocols established by Eli Lilly and the NIH Chemical Genomics Center which tested three assay plates with interleaved high, mid, and low inhibitor concentrations per day for 3 days [44]. Z′ factors ranged from 0.54 to 0.77, which are well above the Z′ factors recommended for cell-based screening [44]. This assay was also amenable to phenotypic multiplexing which provided additional information on cytotoxicity simultaneously with compound potency on human endothelial colony-forming cell (ECFC) migration [44]. Although this stopper-based assay was successfully validated, it is limited for HTS operations because the stopper restricts delivery of reagents to the well and requires manual removal [29,44].

To facilitate the use of automated liquid handling equipment for all steps of the assay, the Oris™ Pro Cell Migration Assays use a self-dissolving biocompatible gel (BCG) spotted in the centers of either 96- or 384-well plates instead of the silicone cell seeding stoppers. The BCG spots act as temporary barriers to prevent cells from settling and attaching during the seeding process and, once dissolved, reveal uniform areas into which cells may migrate [29]. Vogt has demonstrated equivalent performance of the stopper-based and BCG-based assay formats with virtually identical IC_50_ values for latrunculin A of 135 nM and 132 nM, respectively on MDA-MB-231 cells [29]. Gough reported that the Oris™ Pro Cell Migration Assay results in Z′ factors above 0.5 with ECFC cells [44].

The Oris™ Cell Invasion Assays incorporate an ECM overlay in order to form a 3-dimensional environment to study invasion into the cell exclusion zone along the x, y, and z-axes [45]. This assay format provided visual assessment of 3-dimensional invasion of basement membrane extract after 72–96 hours by growth factor stimulated HaCaT II-4 cells in the presence and absence of small molecule signal transduction inhibitors or siRNA knockdown of the plasminogen activator inhibitor-1 gene [45]. This assay format was also used to determine the role of p53 in Snail induced invasion of Hep3B human hepatoma cells. The relative invasion by MSCV-p53WT, p53Mut, and MSCV-Snail infected cells was compared using the Oris™ Cell Invasion Assay with the fluorescence intensity of Calcein AM stained invading cells captured using a microplate reader [46].

Cell exclusion zone assays offer the distinct advantage of not damaging the cells or the ECM as occurs in scratch assays. This assay format also allows continuous visual assessment of the cells throughout the experiment with the ability to acquire multiplexed data unlike the transmembrane assays where the filter restricts observation. In this way, information can be collected regarding morphology, velocity, distance and direction of migrating or invading cells as well as additional phenotypic effects of test compounds. In the Oris™ Cell Invasion Assays, the cell monolayer is entirely surrounded by the ECM thus reflecting a more physiologically relevant environment in which to study cell invasion and the effects of potential cancer therapeutics. In contrast, Brekhman and Neufeld [47] point out that the starting position of the cells for invasion in transmembrane assays is at the liquid-ECM interface which permits only unidirectional invasion; the cells are not embedded between layers of ECM as they are *in vivo*. Cell exclusion zone assays offer robust and reproducible data since the starting dimensions of the detection zone are accurately and precisely positioned in the assay wells. However, these assays are limited for use with adherent cells and cannot be used to establish chemotactic gradients.

## 6. Correlation of Results Between Differing Assay Formats

Several recent studies examined the effects of oncogene expression or signal transduction mechanisms on cell motility in parallel scratch and transmembrane assays and found a good correlation of results. Valster *et al.* [48] demonstrated qualitative agreement on the inhibitory effects of Rac1 siRNA against SNB19 glioma cell invasion through BD BioCoat Matrigel™ invasion chambers and in preventing closure of wounded SNB19 cells in a scratch assay. Similarly, Liu *et al.* [49] found that expression of myristoylated Akt-1 in T4-2 human mammary epithelial cancer cells suppressed their motility in both a scratch assay and for invasion through BD BioCoat Matrigel™ invasion chambers. Kurayoshi and co-workers examined the effects of Wnt-5a on MKN-1 gastric cancer cell migration and invasion [50]. RNA interference against Wnt-5a blocked MKN-1 cell migration and invasion through Matrigel™ coated and uncoated Costar Transwell inserts while anti-Wnt-5a antibodies were effective in blocking MKN-1 cell migration in a scratch assay on a collagen coated coverslip [50]. Bauer *et al.* [51] examined the effects of IGF-I and HGF, alone and in combination, on the ability of L3.6pl pancreatic carcinoma cells to migrate in both BD migration chambers without an ECM coating and scratch assays. IGF-I and HGF each increased the basal level of migration and their combination was additive when tested in both migration assay formats. When tested in BD BioCoat Matrigel™ invasion chambers, these factors acted synergistically [51].

Studies also compared cell exclusion zone assays to either scratch and/or transmembrane assays. Jiang *et al.* [42] demonstrated identical effects of microRNA-138 mediated inhibition of cell migration and invasion in oral tongue squamous cell carcinoma cell lines using the Oris™ Cell Migration Assay and the Cultrex 96-well membrane invasion assay kit from R&D Systems, respectively. Gough *et al.* [44] reported that the Oris™ Cell Migration Assay had fewer steps and yielded results that were more reproducible than the scratch assay. They found that 8 replicates of each drug concentration were necessary in order to generate data in the scratch assay where a sub-standard Z′ factor of 0.20 was achieved. In contrast, the Oris™ Cell Migration Assay required only 4 replicates and achieved a Z′ factor of 0.7. While they could not validate the scratch assay for compound screening because of the high assay variability, they did find an overall correspondence of compound potency data between the two assay formats when a set of compounds that modulate tubulin polymerization or inhibit SRC, RAC1, ROCK1 and myosin II were tested [44].

**Figure 5.**
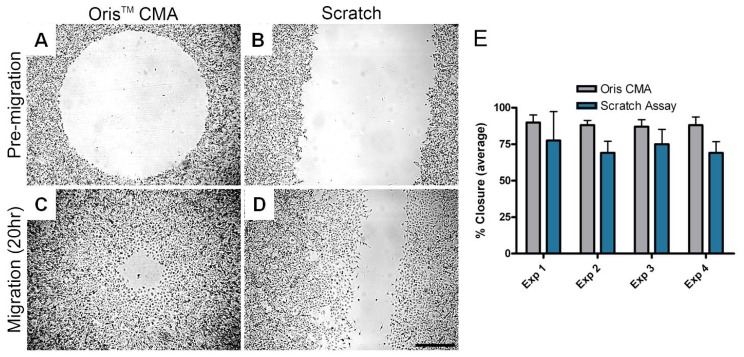
Comparison of MDA-MB-231 human breast epithelial cell migration using the Oris™ Cell Migration Assay and the scratch assay. MDA-MB-231 cells were seeded into collagen I coated Oris™ assay plates with stoppers at 25,000 cells/ 100 μL or in collagen I coated 6-well plates at 500,000 cells/2 mL. Once confluent monolayers were formed, the cells were serum starved for 24 hours. To initiate migration, the stoppers were removed from the Oris™ assay plate and the monolayers were scratched using a 1,000 μL pipet tip in the 6-well plates. The media in both assays was replaced with serum-containing media. Representative phase images of pre-migration (**A** and **C**) and migration after 20 hours (**B** and **D**) in the Oris™ (**A** and **B**) and the scratch (**C** and **D**) assay were captured using a Zeiss Axiovert microscope with an attached CCD camera. Scale bar = 500 μm. Graph of 4 independent experiments comparing cell migration using the Oris™ and scratch assays in parallel (**E**). Images were analyzed using ImageJ analysis software and data presented as average percent closure ± SD (n ≥ 12 replicates).

Researchers at Platypus Technologies compared MDA-MB-231 cell migration on collagen I coated surfaces using both the Oris™ Cell Migration Assay and the scratch assay. Experiments were performed in parallel on four different days to compare the performance of each assay. For each independent experiment, the average area closure achieved using the Oris™ Cell Migration Assay (Figure 5A and B) ranged from 87–90% with a coefficient of variance between 3.7–6.5% (Figure 5E). Conversely, the average area closure obtained using the scratch assay (Figure 5C and D) ranged from 69–77% with a coefficient of variance between 11.3–25.6% (Figure 5E). These results demonstrate that the Oris™ Cell Migration Assay yields more consistent results between experiments with greater reproducibility compared to results obtained using the scratch assay.

There are also examples in which alternate assay formats for studying cell migration or invasion can give different, yet complementary results. Attoub and co-workers [52] used scratch, BD BioCoat Matrigel™ invasion chamber, Oris™ Cell Invasion, and chick heart assays to study the effects of luteolin, a natural product antioxidant that may have histone deacetylase inhibitory activity, on cancer cell invasion. While LNM35 lung cancer cells were shown to migrate in a 24 hour scratch assay and invade in a 24 hour BD BioCoat Matrigel™ invasion chamber, and MCF-7/6 breast cancer cells invaded into embryonic chick heart fragments in an 8-day experiment, neither of these cell lines invaded into the 3-dimensional basement membrane extract in the Oris™ Cell Invasion Assay [52]. In contrast, the highly invasive MDA-MB231-1833 clone did show significant invasion in the Oris™ Cell Invasion Assay after 48 hours. However, invasion of the LNM35, MCF-7/6 and MDA-MB231-1833 cells was reduced by luteolin in a concentration-dependent manner in each of the assay formats [52]. Scott *et al.* [53] examined the effect of LIM kinases 1 and 2 on cell motility using siRNA-mediated knockdown or with a novel small molecule inhibitor (LIMKi) on MDA-MB-231 breast cancer cells. Neither LIMKi nor the siRNAs against LIMK1 and LIMK2, alone or in combination, had any effect on MDA-MB-231 cell migration in an Oris™ Cell Migration Assay [53]. However, the combination of both LIMK1/2 siRNA knockdowns and LIMKi did affect the ability of MDA-MB-231 cells to invade in a modified transmembrane assay. The modified assay entailed inverting Matrigel™ coated Costar Transwell inserts and adhering the cells on the underside of the filters before up righting them in order to allow for cell invasion in Matrigel™. After 5 days, cells were imaged by confocal microscopy at 15 μm intervals. These experiments thus established that LIM kinases are required for 3-dimensional invasion but not for 2-dimensional migration and showed that transmembrane and cell exclusion zone assays may give complementary data on mechanisms of cell motility [53]. Cells in transmembrane invasion assays start out on filters at a liquid-ECM interface [47] which provides a different microenvironment than when the cells are embedded between layers of ECM as they are in Oris™ Cell Invasion Assays. Eccles, Box and Court [4] point out that *in vitro* transmembrane invasion assays favor cells with spindle cell morphology and might be less appropriate for cells with epithelioid morphology. They mention that epithelioid cells might be more dependent on mechanisms of motility that rely upon influences from 3-dimensional microenvironments [4]. Table 1 lists the assay formats available to researchers for the study of cell motility and summarizes the advantages and drawbacks of each.

## 7. Conclusions

There has been a disappointing lack of correlation between compounds that inhibit migration and invasion *in vitro* and their efficacy as cancer therapies in human clinical trials [2,54]. However, use of agents that inhibit migration and invasion, such as matrix metalloproteinase inhibitors, may prove best as chemopreventives since metastases are often already present when cancer is detected in humans [54,55]. Carragher [19] discussed that the limitations of simplistic endpoint assays used to monitor a single target or enzymatic pathway are contributing factors to high attrition rates of drug candidates due to poor efficacy at later stages in the drug discovery process. Assays that target only inhibition of cell motility and disregard other cellular cues may overestimate the clinical activity of the compounds. Therefore, assays that are multiparametric and compatible with automated imaging systems could improve the success of drug discovery efforts by providing more information on compound mechanisms of action.

To accelerate discovery of therapeutics affecting cell motility, cell-based assays should be utilized that:

- Offer ECMs and 3-dimensional environments to mimic cell behavior *in vivo*
- Allow for real-time visualization of cells
- Permit phenotypic and multiparametric analysis of cells (*i.e.*, high-content analysis)
- Facilitate high-throughput screening (automated liquid handling and high content imaging)

When researchers utilize only single target HTS assays, they obtain limited information on how potential therapeutics may influence complex multifaceted events such as tumor cell migration and invasion. As drug discovery screening continues to transition from biochemical to cell-based assays and from high throughput to high content screening, 3-dimensional culture technologies and phenotypic screens will become essential for increasing the relevance of screening assays [18,19]. Profiling individual drug targets by comparing their efficacy in a suite of 3-dimensional migration and invasion models will help determine their utility across a range of tumor invasion mechanisms and allow for a more detailed preclinical evaluation of candidate compounds [4,19]. The miniaturization of motility assays to 384-well formats enables them to be employed earlier in the drug screening paradigm. However, the total assay costs will determine their adoption for use as either primary or secondary screening assays. It remains a challenge to further miniaturize these assays while maintaining a suitable level of robustness that allows their validation for use as high throughput screens. Fortunately, within the assay formats currently available that allow for visual assessment of the cells, multiparametric high content analysis is already a viable option that allows mining of additional phenotypic information and adds immense value with minimal additional cost.

## Figures and Tables

**Table 1.** Summary of Cell Motility Assay Formats.

**Method**	**Advantages**	**Drawbacks**
Scratch Assay Homebrew format: up to 384 wells Commercially available format: 96 wells	Compatible with any configuration of multiwell assay plate Cells move in a defined direction Ability to coat assay surface with a relevant ECM Ability to visually observe cell movement and morphology throughout the experiment Suitable for endpoint and kinetic assays Suitable for multiplexed acquisition of phenotypic data	Methods for creating scratches vary between different labs The size, shape and spacing of the scratches can vary leading to assay variability Difficult to ensure that control and treatment groups of cells are at the same degree of confluence Scratches can damage the underlying ECM Results can be compromised by the release of factors from damaged cells Not suitable for use with non-adherent cells Not suitable for chemotaxis
Transmembrane Assay Commercially available format: 24 or 96 wells	Compatible with adherent and non-adherent cells Permits chemotaxis Ability to coat assay surface with a relevant ECM Cells move in a defined direction	Assay requires many steps to set up Chemotactic gradient is non-linear Difficult to visualize the cells and observe morphology Not suitable for kinetic assays Difficult to obtain accurate and statistically significant results as only a small number of cells cross through the membrane Difficult to enumerate cells when their distribution and/or staining is uneven
Microfluidics Assay Commercially available format: 96 or 192 wells	Assay chambers may be filled with relevant ECM Assays require low numbers of cells and low volumes of reagents Compatible with adherent and non-adherent cells Permits chemotaxis Ability to visually observe cell movement and morphology throughout the experiment Cells move in a defined direction Suitable for endpoint and kinetic assays Suitable for multiplexed acquisition of phenotypic data	Assays can be technically challenging to perform Evaporation is a concern Daily exchanges of growth media/inhibitors is required thus increasing hands-on time and labor involved in the assay Interfaces must be developed to allow for assay automation and ease of handling Materials used in fabricating the microfluidic arrays must be compatible with critical control of cell microenvironments yet be cost effective
Cell Exclusion Zone Assay Commercially available format: 24, 96 or 384 wells	No damage to cells or ECM Ability to coat assay surface with a relevant ECM Ability to visually observe cell movement and morphology throughout the experiment Cells move in a defined direction Suitable for endpoint and kinetic assays Suitable for multiplexed acquisition of phenotypic data Cells are 3-dimensionally embedded in ECM in the invasion assay format	Not suitable for use with non-adherent cells Not suitable for chemotaxis
